# Ergodic Rate Analysis of Simultaneous Transmitting and Reflecting Reconfigurable Intelligent Surface-Assisted Rate-Splitting Multiple Access Systems Based on Discrete Phase Shifts

**DOI:** 10.3390/s24175480

**Published:** 2024-08-23

**Authors:** Tao Liu, Yue Zhou

**Affiliations:** College of Electronic and Information Engineering, Nanjing University of Aeronautics and Astronautics, Nanjing 210016, China; yzhou2023@nuaa.edu.cn

**Keywords:** rate-splitting multiple access, simultaneous transmitting and reflecting reconfigurable intelligent surface, discrete phase shift, ergodic rate

## Abstract

In this paper, we combine simultaneous transmitting and reflecting reconfigurable intelligent surface (STAR-RIS) with rate-splitting multiple access (RSMA) technology and investigate the ergodic rate performance of an STAR-assisted RSMA system. Considering the discrete phase shifts of the STAR-RIS in practice, the downlink performance of STAR-RIS-assisted RSMA with discrete phase shifts is compared to that with continuous phase shifts. Firstly, the cumulative distribution function of signal-to-interference-plus-noise ratio (SINR) of users is analyzed. Then, the total ergodic rate of the system and its approximate closed-form solution are, respectively, derived based on the cumulative distribution function of users. The simulation results validate the effectiveness of the theoretical analysis, showing good agreement between the derived theoretical ergodic rate and the corresponding simulations. Although the system performance with discrete phase shifts is inferior to that with continuous phase shifts due to quantization errors, the performance of the continuous phase shift system is well approximated when the quantization bit of the phase shift system reaches 3 in the simulations. Additionally, the impact of the number of STAR-RIS elements on the system’s performance is analyzed.

## 1. Introduction

The development of communication technology has always been a key factor driving progress in modern society. From early telegraphs and telephones to modern mobile communication and the internet, each technological leap has greatly changed people’s lifestyles and the way that information is disseminated. Since the advent of traditional wireless communication, people have always believed that the propagation medium is a physical entity of random behavior between transmitters and receivers [1]. The interaction of uncontrollable electromagnetic waves with objects between transmitters and receivers degrades the quality of received signals. In recent years, with the development of smart surface technology, the field of communication has again undergone revolutionary changes. Reconfigurable intelligent surfaces (RISs) are an emerging communication technology that dynamically adjust the propagation characteristics of signals by deploying a large number of passive reflecting elements on the wireless signal propagation path [2]. This optimization of the signal transmission path enhances communication quality, extends signal coverage, and reduces signal interference. The potential advantages of RIS technology lie in its flexibility and programmability.

In addition, notable potential features of the RIS include energy efficiency and cost effectiveness. Its reflecting elements only passively reflect the impinging signals, without requiring any transmit radio frequency (RF) chains, and thus can be operated with orders-of-magnitude-lower energy costs compared to traditional relays and amplifiers [3,4]. The RIS is able to optimize signal transmission paths by passively reflecting and actively adjusting the electromagnetic wave propagation environment, thereby reducing signal attenuation and interference in conventional systems. Moreover, the flexible deployment features of the RIS enable it to work in key locations within the network, to cover blind or weak signal areas. The RIS’s capabilities allow communication systems to achieve the same coverage and data rates at lower transmit power, directly reducing energy consumption [5].

With further research, scholars have proposed a simultaneous transmitting and reflecting reconfigurable intelligent surface (STAR-RIS) as a branch of RIS technology. This surface can switch between transmitting and reflecting, expanding the functionality of intelligent surfaces. STAR-RIS not only reflects signals to enhance signal strength but also transmits signals, allowing signals to pass through the surface for further propagation. This feature is particularly useful for improving signal penetration and coverage range, especially in densely built urban environments, where signals may attenuate due to obstacles like buildings [6]. By deploying an STAR-RIS, it is possible to effectively bypass obstacles and achieve continuous signal coverage.

Mobile communication technology has undergone several technological innovations since its inception, with multiple access technology being key to enabling multi-user communication. Orthogonal multiple access (OMA) was a widely used technology in early mobile communication systems; it ensured signal orthogonality by assigning unique time or frequency resources to each user to avoid interference. However, with an increasing number of users, spectrum scarcity becomes more pronounced. To address this issue, non-orthogonal multiple access (NOMA) has emerged. NOMA allows multiple users to share the same time-frequency resources, distinguishing users within a power domain to achieve multi-user access [7,8]. NOMA leverages the different channels between users and employs successive interference cancelation (SIC) at the receiver to decode interference completely, thereby reducing interference among multiple users. This technology enhances spectrum efficiency, enabling more users to communicate using the same resources. Compared to NOMA, which is suitable for scenarios with strong multi-user interference, rate-splitting multiple access (RSMA) has broader applicability. RSMA further optimizes the way multiple users share resources by splitting each user’s data streams into multiple sub-streams; independently coding, modulating, and allocating power to these sub-streams; and, after that, combining all the sub-streams into a composite signal for transmission [9]. This technology not only improves spectrum efficiency but also enhances system flexibility and fairness, enabling dynamic resource allocation based on users’ real-time requirements.

In [10,11,12], a detailed study on RSMA technology was provided. The principles and communication models of RSMA were introduced in reference [10], which compared and summarized the advantages and disadvantages of several multiple access technologies including OMA, NOMA, and RSMA in communication systems. It pointed out that the signal interference issue in 5G and 6G had the greatest impact on key technical indicators in communication systems. The relationship between RSMA, spatial division multiple access (SDMA), and NOMA was clarified in [11]. It proposed that RSMA acted as a bridge between SDMA and NOMA. SDMA treated interference completely as noise, whereas NOMA decoded interference entirely. Meanwhile, RSMA decoded part of the interference and treated part as noise. The combination of cooperative user relaying (CUR) with RSMA in wireless communication networks was explored in reference [12], which discussed the core challenges of CUR and RSMA and looked ahead to the prospects of their combination. Due to the promising future of RSMA and RIS, research on RIS and RSMA has attracted widespread attention. Reference [13] studied a wired and tethered unmanned aerial vehicle (UAV) communication system assisted by RIS and optimized the UAV position, RIS phase, and RSMA parameters to maximize the weighted sum-rate of users. In [14], a quantum machine learning (QML) method was proposed to maximize the energy efficiency of RIS-assisted RSMA communications. Compared to conventional optimization, QML achieved the same optimization performance with lower complexity. A hybrid airborne full-duplex (FD) relay system with a reconfigurable intelligent surface on the UAV was considered in [15]. Finite block length encoding RSMA was used to improve spectral efficiency and reduce latency. Reference [15] also employed the alternating optimization (AO) method to handle the non-convex problem of the weighted sum-rate maximization. The ergodic capacity in a multi-user RSMA-RIS communication system was investigated in [16], which compared RSMA-RIS with NOMA-RIS and showed that RSMA-RIS outperformed in both single-user ergodic capacity and multi-user average ergodic capacity.

Combining STAR-RIS with RSMA, for green communication and expanded communication coverage, has been studied in various papers. Closed-form solutions for user outage probability under Energy Splitting (ES) and Mode Switching (MS) STAR-RIS protocols were provided in [17]. Reference [17] also demonstrated the potential of RSMA and STAR-RIS technologies to enhance next-generation wireless communication networks. Reference [18] investigated spatially correlated Rayleigh channels in RSMA-assisted STAR-RIS communication, deriving new expressions for joint moments of spatially correlated Rayleigh channels. In [19], covert communication issues in an STAR-RIS-assisted RSMA system were explored. It highlighted the impact of STAR-RIS’s element reflectivity on covert performance relative to transmit power. When the transmit power was high enough, the detection error probability was a monotonically increasing function of the reflection coefficient. The potential of combining STAR-RIS with RSMA for assisting multi-device communication was underscored in [20], which achieved significant performance improvements through parameter adjustments. Although there exists a large number of studies on STAR-RIS and RSMA communication, they lack consideration of the discrete phase shifts of STAR-RIS, which is not realistic. Based on this, the main contributions of this paper are as follows.

(1) We primarily consider a multi-user downlink RSMA communication system assisted by STAR-RIS. Firstly, we cluster the multiple users and ignore the interference between different user clusters. Secondly, considering that users are uniformly distributed within the range centered on the STAR-RIS, we quantify the phase shifts of the STAR-RIS. With this result, we further derive the theoretical values of the system ergodic rate under both the discrete and continuous phase shifts of the STAR-RIS for the performance evaluation.
(2) Through Monte Carlo simulations, we verify the correctness of the theoretical derivations and demonstrate that the system’s ergodic rate with the discrete phase shifts of the STAR-RIS is inferior to that with the continuous phase shifts. However, increasing the number of quantization bits can make the performance of the discrete phase shifts approach that of the continuous phase shifts. Additionally, we show the impact of the number of STAR-RIS elements, the path loss exponent, and the different modes of STAR-RIS on the system performance.

## 2. System Model

### 2.1. Channel Model

**Figure 1:**
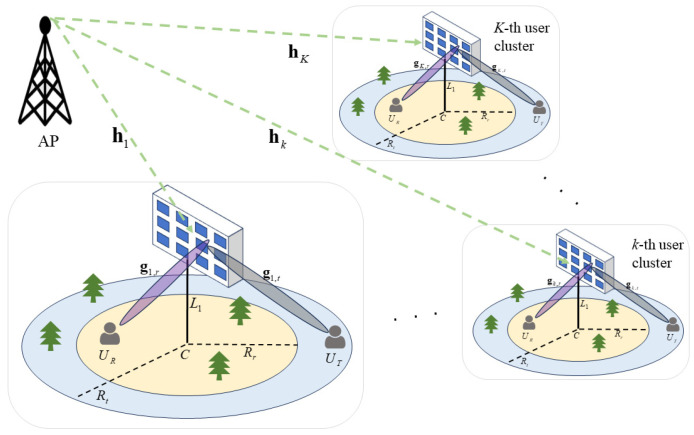
System model of STAR-RIS.

In the STAR-RIS-assisted downlink RSMA system model, there is one wireless AP, *K* STAR-RIS units, and 2*K* users. As shown in Figure 1, each STAR-RIS consists of *N* elements, and both the AP and the users are equipped with only one antenna each. The 2*K* users are divided into *K* user clusters, each containing one STAR-RIS, with the transmitting user and reflecting user located on opposite sides of the STAR-RIS. It is assumed that the direct channels between the AP and the users are blocked by obstacles [21,22].

In this paper, it is assumed that there is a block between individual user clusters, so that the mutual influence between user clusters can be ignored. The channel between the AP and STAR-RIS is modeled as a Rayleigh channel, denoted by $h_{k}=\sqrt{\rho_{0}/d_{AP,k}^{\alpha }}{\tilde{h}}_{k}$. In the statement, $\rho_{0}$ denotes the path loss at a reference unit distance, α is the path loss exponent, $d_{AP,k}$ refers to the distance between the AP and the *k*-th STAR-RIS, and the *n*-th element in ${\tilde{h}}_{k}\in {\mathbb{C}}^{N\times 1}$ satisfies ${\tilde{h}}_{k,n}∼CN\left(0,1\right)$. Due to the random distribution of the transmitting user T and the reflecting user R within a circular area, with the center point *C*, the STAR-RIS is located meters above point *C*. It is also assumed that the STAR-RIS can obtain the channel state information of the users. The STAR-RIS to the corresponding user is also modeled as a Rayleigh channel, denoted as $g_{k,m}=\sqrt{\rho_{0}/{\left(d_{k,m}^{2}+L_{k}^{2}\right)}^{\alpha /2}}{\tilde{g}}_{k,m}$, where $d_{k,m}$ denotes the distance from user *m* to the center point *C*, and ${\tilde{g}}_{k,m,n}$ is the *n*-th element in ${\tilde{g}}_{k,m}$ and satisfies ${\tilde{g}}_{k,m,n}∼CN\left(0,1\right)$.$m\in \left\{t,r\right\}$ denotes the type of user in the *k*-th user cluster, m=t denotes the transmitting user, and m=r is the reflecting user.

Since the user clusters are independent of each other, this paper specifically analyzes a single user cluster. It is assumed that the reflecting users are near users with better channel gains, and the transmitting users are far users. The positions of the users are modeled using a homogeneous Poisson point process [23]. The reflecting users are distributed within a circle of radius $R_{1}$, with polar coordinate $\left(d_{k,r},\vartheta_{k,r}\right)$. The transmitting users are distributed within an annular region between radius $R_{1}$ and radius $R_{2}$, where $R_{1}<R_{2}$, with polar coordinate $\left(d_{k,t},\vartheta_{k,t}\right)$. The probability density function for the distance from the users to the center point and the angle is as follows [23].

(1) $$f_{d_{k,r}}\left(x\right)=\frac{\partial }{\partial x}\frac{\pi x^{2}}{\pi R_{1}^{2}}=\frac{2x}{R_{1}^{2}}\;\;\;0<x<R_{1},$$

(2) $$f_{d_{k,t}}\left(x\right)=\frac{\partial }{\partial x}\frac{\pi \left(x^{2}-R_{1}^{2}\right)}{\pi \left(R_{2}^{2}-R_{1}^{2}\right)}=\frac{2x}{R_{2}^{2}-R_{1}^{2}}\;\;\;R_{1}<x<R_{2},$$

(3) $$f_{\vartheta_{k,l}}\left(x\right)=\frac{1}{\pi }\;\;\;\;-\frac{\pi }{2}<x<\frac{\pi }{2}.$$

### 2.2. STAR-RIS-Assisted RSMA Downlink Communication Model

Based on the working principle of the STAR-RIS, the signal is transmitted to the user T through the transmission module of the STAR-RIS and to the user R through the reflection module. The STAR-RIS works in the ES mode. Assuming that all elements on the STAR-RIS have the same amplitude coefficients, the transmission factor and reflection factor matrices for the *k*-th user cluster are, respectively, represented as

(4) $$\Theta_{k,t}=\sqrt{\beta_{k,t}}diag\left(e^{j\theta_{k,t,1}},e^{j\theta_{k,t,2}},\cdots ,e^{j\theta_{k,t,N}}\right),$$

(5) $$\Theta_{k,r}=\sqrt{\beta_{k,r}}diag\left(e^{j\theta_{k,r,1}},e^{j\theta_{k,r,2}},\cdots ,e^{j\theta_{k,r,N}}\right),$$

where $\beta_{k,t}$ and $\beta_{k,r}$ represent the transmission and reflection amplitude coefficients in the *k*-th user cluster, respectively, and satisfy $\beta_{k,t}+\beta_{k,r}\leq 1$, $\beta_{k,t},\beta_{k,r}\in \left[0,1\right]$ [22]. The phase shift is $\theta_{n}^{k,t}$, $\theta_{n}^{k,r}\in \left[0,2\pi \right)$ and $n\in \left\{1,2\ldots ,N\right\}$.

For the RSMA-assisted communication system, all the common messages are encoded into a single common stream which must be decoded by all users, and all the private messages are encoded into individual private streams which will be decoded by their respective receivers. By dividing the information into private and common parts, RSMA can make more efficient use of the available spectrum, especially if there is interference between users. RSMA can flexibly adjust the ratio of private and common signals according to channel conditions and user requirements, thus adapting to different network environments. Based on this, the transmitting signals are classified into private and common signals, and the transmitting signal at the AP can be represented as

(6) $$x=\sqrt{a_{c}p_{s}}s_{c}+\sum_{k=1}^{K}\sqrt{a_{k,t}^{p}p_{s}}s_{k,t}^{p}+\sum_{k=1}^{K}\sqrt{a_{k,r}^{p}p_{s}}s_{k,r}^{p},$$

where $p_{s}$ is the total power of the signal transmitted by the AP. $s_{c}$, $s_{k,t}^{p}$, and $s_{k,r}^{p}$ are the common information and the private information of user T and user R in the *k*-th user cluster. The power allocation coefficients of the common and private information are denoted by $a_{c}$ and $a_{k,m}^{p}$, respectively, and satisfy $a_{c}+\sum_{k=1}^{K}\left(a_{k,t}^{p}+a_{k,r}^{p}\right)=1$.

This gives a receiving signal at user *m* in the *k*-th user cluster of

(7) $$y_{k,m}={\left(g_{k,m}\right)}^{H}\Theta_{k,m}h_{k}x+n_{k,m},$$

where $n_{k,m}$ is the additive Gaussian white noise of user *m*, obeying $n_{k,m}∼CN\left(0,\sigma_{n}^{2}\right)$.

According to the decoding order of RSMA, user *m* of the *k*-th user cluster will decode the common information first, and its signal-to-interference-plus-noise ratio (SINR) is

(8) $$\gamma_{k,m}^{c}=\frac{a_{c}p_{s}{\left|{\left(g_{k,m}\right)}^{H}\Theta_{k,m}h_{k}\right|}^{2}}{p_{s}\left(\sum_{k=1}^{K}\left(a_{k,t}^{p}+a_{k,r}^{p}\right)\right){\left|{\left(g_{k,m}\right)}^{H}\Theta_{k,m}h_{k}\right|}^{2}+\sigma_{n}^{2}}.$$

After performing SIC on common information, the user detects their own private information. The remaining interference consists only of other users’ private information. The SINR for user *m* in the *k*-th user cluster is

(9) $$\gamma_{k,m}^{p}=\frac{a_{k,p}^{m}p_{s}{\left|{\left(g_{k,m}\right)}^{H}\Theta_{k,m}h_{k}\right|}^{2}}{p_{s}\left(\sum_{i=1,i\neq k}^{K}\left(a_{i,m}^{p}\right)+\sum_{i=1}^{K}a_{i,\bar{m}}^{p}\right){\left|{\left(g_{k,m}\right)}^{H}\Theta_{k,m}h_{k}\right|}^{2}+\sigma_{n}^{2}},$$

in which $\bar{m}\in \left\{t,r\right\}$. When m=t, $\bar{m}=r$, and vice versa.

## 3. Ergodic Rate Analysis

### 3.1. Ergodic Rate under Discrete Phase Shifts

It is assumed that the STAR-RIS has known the instantaneous channel state information [20], which was obtainable through popular channel estimation techniques such as parallel factor decomposition. The parameters of STAR-RIS can be adjusted to maximize the SINR for achieving the highest ergodic rate. Considering that the transmitter has a single antenna, let the *n*-th element in $g_{k,m}$ be $g_{k,m,n}=\left|g_{k,m,n}\right|e^{j\phi_{k,m,n}}$ and the *n*-th element in $h_{k}$ be $h_{k,n}=\left|h_{k,n}\right|e^{j\varphi_{k,n}}$. The optimal phase $\theta_{k,m,n}$ of the STAR-RIS is $\theta_{k,m,n}^{*}=\phi_{k,m,n}-\varphi_{k,n}$. Due to the actual hardware limitation, $\theta_{k,m,n}$ can only take a limited number of discrete values, represented by the set $S=\left\{0,\Delta \theta ,\ldots ,\left(2^{b}-1\right)\Delta \theta \right\}$, where $\Delta \theta =2\pi /2^{b}$ and *b* represents the number of quantization bits. The optimal discrete phase shift variable is then represented as [24]

(10) $${\bar{\theta }}_{k,m,n}=\Delta \theta \left\lfloor \frac{\theta_{k,m,n}^{*}}{\Delta \theta }+\frac{1}{2}\right\rfloor .$$

From the above equation, it can be seen that the phase quantization error is $\theta_{k,m,n}^{e}={\bar{\theta }}_{k,m,n}-\theta_{k,m,n}^{*}$, which obeys the uniform distribution of the interval on $\left[-\Delta \theta /2,\Delta \theta /2,\right]$. According to the central limit theorem (CLT), when N≫1, $X_{k,m}=\left(\sum_{n=1}^{N}\left|{\tilde{h}}_{k,n}\right|\left|{\tilde{g}}_{k,n}^{m}\right|e^{j\theta_{k,m,n}^{e}}\right)/N$ obeys the complex Gaussian distribution, such that its real part $U=ℜ\left\{X_{k,m}\right\}$ and the imaginary part $V=ℑ\left\{X_{k,m}\right\}$. The real and imaginary parts are independent of each other and obey $U∼N\left(\mu ,\sigma_{U}^{2}\right)$ and $V∼N\left(0,\sigma_{V}^{2}\right)$, and there are the following relationships according to [25].

(11) $$\mu =\varphi_{1}\mu_{0},$$

(12) $$\sigma_{U}^{2}=\frac{1}{2N}\left(1+\varphi_{2}-2\varphi_{1}^{2}\mu_{0}^{2}\right),$$

(13) $$\sigma_{V}^{2}=\frac{1}{2N}\left(1-\varphi_{2}\right).$$

In Equations (11)–(13), $\varphi_{p}=E\left\{e^{jp\theta_{k,m,n}^{e}}\right\}$ is the characteristic function. Since the probability density function of $\theta_{k,m,n}^{e}$ is symmetric about the vertical axis, the calculation yields $\varphi_{1}=Sa\left(\Delta \theta /2\right)$ and $\varphi_{2}=Sa\left(\Delta \theta \right)$, where $Sa\left(x\right)=\sin\left(x\right)/x$ is the sampling function and $\mu_{0}$ is the mean of $\left(\sum_{n=1}^{N}\left|{\tilde{h}}_{k,n}\right|\left|{\tilde{g}}_{k,n}^{m}\right|\right)/N$ with value $\mu_{0}=\pi /4$.

Under conditions of N≫1 and $\varphi_{1}>0$, the distribution of $\left|X_{k,m}\right|=\sqrt{U^{2}+V^{2}}$ can be approximated by the Nakagami-*m* distribution [25]. Denote $Z_{k,m}={\left|X_{k,m}\right|}^{2}$, then $Z_{k,m}$ obeys the gamma distribution and its cumulative distribution function is

(14) $$F_{Z_{k,m}}\left(z\right)=\frac{\gamma \left(\lambda ,\frac{\lambda }{\Omega }z\right)}{\Gamma \left(\lambda \right)},$$

where $\Omega =\mu^{2}$, $\lambda =N\varphi_{1}^{2}\mu_{0}^{2}/\left(1+\varphi_{2}-2\varphi_{1}^{2}\mu_{0}^{2}\right)/2$ and $\gamma \left(s,x\right)$ is the lower incomplete gamma function [26]. The discrete phase shift matrix can be substituted into (8) and (9) and they can be simplified to obtain the SINR for the user to decode the common and private information as

(15) $$\gamma_{k,m}^{c}=\frac{\tau_{k,m}a_{c}Z_{k,m}}{\tau_{k,m}\left(1-a_{c}\right)Z_{k,m}+{\left(d_{k,m}^{2}+L_{k}^{2}\right)}^{\alpha /2}},$$

(16) $$\gamma_{k,m}^{p}=\frac{\tau_{k,m}a_{k,m}^{p}Z_{k,m}}{\tau_{k,m}\left(1-a_{c}-a_{k,m}^{p}\right)Z_{k,m}+{\left(d_{k,m}^{2}+L_{k}^{2}\right)}^{\alpha /2}},$$

where there is $\tau_{k,m}=N^{2}\rho_{0}^{2}\beta_{k,m}p_{s}/\left(\sigma_{n}^{2}d_{AP,k}^{\alpha }\right)$. The ergodic rate of the common and private information of user *m* in the *k*-th user cluster can be calculated using the Shannon equation,

(17) $$\begin{array}{l} {\bar{R}}_{k,m}^{\chi }=E\left\{\log_{2}\left(1+\gamma_{k,m}^{\chi }\right)\right\}\\ =\frac{1}{\ln2}\int_{0}^{\infty }\frac{1-F_{k,m}^{\chi }\left(x\right)}{1+x}dx \end{array},$$

where $\chi \in \left\{c,p\right\}$ represents the rate of common and private messages sent by the corresponding user, respectively, and $F_{k,m}^{\chi }\left(x\right)$ denotes the cumulative distribution function of $\gamma_{k,m}^{\chi }$. Substituting Equations (1)–(3) and (14) into (17), the ergodic rate of common and private messages of user *m* can be further derived as

(18) $${\bar{R}}_{k,m}^{\chi }=\frac{1}{\ln2}\int_{-\pi /2}^{\pi /2}\int_{\delta_{1}}^{\delta_{2}}\int_{0}^{\varpi_{k,m}^{\chi }}\frac{1-F_{Z_{k,m}}\left({Τ}_{k,m}^{\chi }\left(x\right)\Psi \left(y\right)\right)}{1+x}f_{d_{k,m}}\left(y\right)f_{\vartheta_{k,m}}\left(z\right)dxdydz.$$

In the formula, there exist ${Τ}_{k,m}^{\chi }\left(x\right)=x/\left(v_{k,m}^{\chi }-u_{k,m}^{\chi }x\right)$ and $\Psi \left(y\right)={\left(y^{2}+L_{k}^{2}\right)}^{\alpha /2}$. For the reflecting user, $\delta_{1}=0$ and $\delta_{2}=R_{1}$. The expressions for the common information rate are $v_{k,r}^{c}=\tau_{k,r}a_{c}$, $u_{k,r}^{c}=\tau_{k,r}\left(1-a_{c}\right)$ and $\varpi_{k,r}^{c}=a_{c}/\left(1-a_{c}\right)$. The expressions for the private information rate are $v_{k,r}^{p}=\tau_{k,r}a_{k,r}^{p}$, $u_{k,r}^{p}=\tau_{k,r}\left(1-a_{c}-a_{k,r}^{p}\right)$ and $\varpi_{k,r}^{c}=a_{k,r}^{p}/\left(1-a_{c}-a_{k,r}^{p}\right)$. For the transmitting user, $\delta_{1}=R_{1}$ and $\delta_{2}=R_{2}$. The common information rate is calculated as $v_{k,t}^{c}=\tau_{k,t}a_{c}$, $u_{k,t}^{c}=\tau_{k,t}\left(1-a_{c}\right)$ and $\varpi_{k,t}^{c}=a_{c}/\left(1-a_{c}\right)$, and the private information rate is calculated as $v_{k,t}^{p}=\tau_{k,t}a_{k,t}^{p}$, $u_{k,t}^{p}=\tau_{k,t}\left(1-a_{c}-a_{k,t}^{p}\right)$ and $\varpi_{k,r}^{c}=a_{k,t}^{p}/\left(1-a_{c}-a_{k,t}^{p}\right)$. The calculation of the triple integral is

(19) $$\begin{array}{l} {\bar{R}}_{k,m}^{\chi }=\frac{1}{\ln2}\int_{\delta_{1}}^{\delta_{2}}\int_{0}^{\varpi_{k,m}^{\chi }}\frac{1-F_{Z_{k,m}}\left({Τ}_{k,m}^{\chi }\left(x\right)\Psi \left(y\right)\right)}{1+x}f_{d_{k,m}}\left(y\right)dxdy\\ =\underset{{Ι}_{1}}{\underset{︸}{\frac{1}{\ln2}\int_{\delta_{1}}^{\delta_{2}}\int_{0}^{\varpi_{k,m}^{\chi }}\frac{f_{d_{k,m}}\left(y\right)}{1+x}dxdy}}-\underset{{Ι}_{2}}{\underset{︸}{\frac{1}{\ln2}\int_{\delta_{1}}^{\delta_{2}}\int_{0}^{\varpi_{k,m}^{\chi }}\frac{F_{Z_{k,m}}\left({Τ}_{k,m}^{\chi }\left(x\right)\Psi \left(y\right)\right)}{1+x}f_{d_{k,m}}\left(y\right)dxdy}} \end{array}.$$

Calculating the results of terms ${Ι}_{1}$ and ${Ι}_{2}$ separately, it can be obtained that

(20) $$\begin{array}{l} {Ι}_{1}=\frac{1}{\left(\delta_{2}^{2}-\delta_{1}^{2}\right)\ln2}\int_{\delta_{1}}^{\delta_{2}}2y\int_{0}^{\varpi_{k,m}^{\chi }}\frac{1}{1+x}dxdy\\ =\log_{2}\left(1+\varpi_{k,m}^{\chi }\right) \end{array},$$

(21) $$\begin{array}{l} {Ι}_{2}=\frac{\varpi_{k,m}^{\chi }\pi^{2}}{2\ln2\left(\delta_{2}+\delta_{1}\right)N_{p}^{2}Τ\left(\lambda \right)}\sum_{i=1}^{N_{p}}\sum_{j=1}^{N_{p}}\left\{\frac{\left(\frac{\delta_{2}-\delta_{1}}{2}v_{j}+\frac{\delta_{2}+\delta_{1}}{2}\right)\sqrt{\left(1-v_{i}^{2}\right)\left(1-v_{j}^{2}\right)}}{\left(1+\varpi_{k,m}^{\chi }\left(v_{i}+1\right)/2\right)}\times \right.\\ \left.\gamma \left(\lambda ,\frac{\lambda }{\Omega }{Τ}_{k,m}^{\chi }\left(\varpi_{k,m}^{\chi }\left(1+v_{i}\right)/2\right)\Psi \left(\frac{\delta_{2}-\delta_{1}}{2}v_{j}+\frac{\delta_{2}+\delta_{1}}{2}\right)\right)\right\} \end{array}.$$

The specific calculation of ${Ι}_{2}$ is expanded. Firstly, the cumulative distribution function of $Z_{k,m}$ is substituted into the ${Ι}_{2}$ term of (19) with the expression

(22) $${Ι}_{2}=\frac{2}{\ln2\left(\delta_{2}^{2}-\delta_{1}^{2}\right)\Gamma \left(\lambda \right)}\int_{\delta_{1}}^{\delta_{2}}\int_{0}^{\varpi_{k,m}^{\chi }}\frac{y}{1+x}\gamma \left(\lambda ,\frac{\lambda }{\Omega }{Τ}_{k,m}^{\chi }\left(x\right)\Psi \left(y\right)\right)dxdy.$$

Subsequently, a change of variables is performed for the inner integral and then the Chebyshev–Gauss quadrature formula is used for approximation, obtaining

(23) $$\begin{array}{l} \int_{0}^{\varpi_{k,m}^{\chi }}\frac{y}{1+x}\gamma \left(\lambda ,\frac{\lambda }{\Omega }{Τ}_{k,m}^{\chi }\left(x\right)\Psi \left(y\right)\right)dx\\ =\frac{\varpi_{k,m}^{\chi }}{2}\int_{-1}^{1}\frac{y}{1+\varpi_{k,m}^{\chi }\left(x+1\right)/2}\gamma \left(\lambda ,\frac{\lambda }{\Omega }{Τ}_{k,m}^{\chi }\left(\varpi_{k,m}^{\chi }\left(x+1\right)/2\right)\Psi \left(y\right)\right)dx\\ =\frac{\varpi_{k,m}^{\chi }\pi }{2N_{p}}\sum_{i=1}^{N_{p}}\frac{y\sqrt{1-v_{i}^{2}}}{1+\varpi_{k,m}^{\chi }\left(v_{i}+1\right)/2}\gamma \left(\lambda ,\frac{\lambda }{\Omega }{Τ}_{k,m}^{\chi }\left(\varpi_{k,m}^{\chi }\left(v_{i}+1\right)/2\right)\Psi \left(y\right)\right) \end{array},$$

where $v_{i}=\cos\left(\frac{2i-1}{2N_{p}}\pi \right)$.

Thus, ${Ι}_{2}$ can be converted into a single integral with the expression

(24) $${Ι}_{2}=\frac{\varpi_{k,m}^{\chi }\pi }{\ln2\left(\delta_{2}^{2}-\delta_{1}^{2}\right)\Gamma \left(\lambda \right)N_{p}}\sum_{i=1}^{N_{p}}\underset{{Ι}_{3}}{\underset{︸}{\int_{\delta_{1}}^{\delta_{2}}\frac{y\sqrt{1-v_{i}^{2}}}{1+\varpi_{k,m}^{\chi }\left(v_{i}+1\right)/2}\gamma \left(\lambda ,\frac{\lambda }{\Omega }{Τ}_{k,m}^{\chi }\left(\varpi_{k,m}^{\chi }\left(v_{i}+1\right)/2\right)\Psi \left(y\right)\right)}}dy.$$

Processing ${Ι}_{3}$ using a similar method of Chebyshev–Gauss quadrature, the expression can be rewritten as

(25) $$\begin{array}{l} {Ι}_{3}=\int_{\delta_{1}}^{\delta_{2}}\frac{y\sqrt{1-v_{i}^{2}}}{1+\varpi_{k,m}^{\chi }\left(v_{i}+1\right)/2}\gamma \left(\lambda ,\frac{\lambda }{\Omega }{Τ}_{k,m}^{\chi }\left(\varpi_{k,m}^{\chi }\left(v_{i}+1\right)/2\right)\Psi \left(y\right)\right)dy\\ =\frac{\left(\delta_{2}-\delta_{1}\right)}{2}\int_{-1}^{1}\left\{\frac{\left(\left(\delta_{2}-\delta_{1}\right)t/2+\left(\delta_{2}+\delta_{1}\right)/2\right)\sqrt{1-v_{i}^{2}}}{\left(1+\varpi_{k,m}^{\chi }\left(v_{i}+1\right)/2\right)}\right.\times \\ \left.\gamma \left(\lambda ,\frac{\lambda }{\Omega }{Τ}_{k,m}^{\chi }\left(\varpi_{k,m}^{\chi }\left(v_{i}+1\right)/2\right)\Psi \left(\left(\delta_{2}-\delta_{1}\right)t/2+\left(\delta_{2}+\delta_{1}\right)/2\right)\right)\right\}dt\\ =\frac{\left(\delta_{2}-\delta_{1}\right)\pi }{2N_{p}}\sum_{j=1}^{N_{p}}\left\{\frac{\left(\left(\delta_{2}-\delta_{1}\right)v_{j}/2+\left(\delta_{2}+\delta_{1}\right)/2\right)\sqrt{1-v_{i}^{2}}\sqrt{1-v_{j}^{2}}}{\left(1+\varpi_{k,m}^{\chi }\left(v_{i}+1\right)/2\right)}\right.\times \\ \left.\gamma \left(\lambda ,\frac{\lambda }{\Omega }{Τ}_{k,m}^{\chi }\left(\varpi_{k,m}^{\chi }\left(v_{i}+1\right)/2\right)\Psi \left(\left(\delta_{2}-\delta_{1}\right)v_{j}/2+\left(\delta_{2}+\delta_{1}\right)/2\right)\right)\right\} \end{array},$$

where $v_{j}=\cos\left(\frac{2j-1}{2N_{p}}\pi \right)$.

The conclusion of Equation (21) is obtained by collating Equations (24) and (25). Finally, substituting Equations (20) and (21) into Equation (19) yields an approximate closed expression for ${\bar{R}}_{k,m}^{\chi }$.

### 3.2. Ergodic Rate under Continuous Phase Shifts

When STAR-RIS has continuous phase shifts, the value of the optimal phase $\theta_{k,m,n}$ is $\theta_{k,m,n}^{*}=\phi_{k,m,n}-\varphi_{k,n}$. Since the channels of AP to STAR-RIS and STAR-RIS to users are independent of each other and both ${\tilde{h}}_{k}$ and ${\tilde{g}}_{k,n}^{m}$ obey a complex Gaussian distribution with mean 0 and variance 1,$\left|{\tilde{h}}_{k,n}\right|$ and $\left|{\tilde{g}}_{k,n}^{m}\right|$ obey a Rayleigh distribution. Let $X_{k}=\sum_{n=1}^{N}\left|{\tilde{h}}_{k,n}\right|\left|{\tilde{g}}_{k,n}^{m}\right|$; according to the CLT, it can be obtained that $X_{k}∼N\left(N\mu_{0},N\sigma_{0}^{2}\right)$, where $\mu_{0}=\frac{\sqrt{\pi }}{2}\times \frac{\sqrt{\pi }}{2}=\frac{\pi }{4}$ and $\sigma_{0}^{2}=1-\pi^{2}/16$ [27]. Let $Y_{k,m}={\left(\sum_{n=1}^{N}\left|{\tilde{h}}_{k,n}\right|\left|{\tilde{g}}_{k,n}^{m}\right|\right)}^{2}/N\sigma_{0}^{2}$; then, $Y_{k,m}$ obeys the non-central chi square distribution of $Y_{k,m}∼\chi_{1}^{2}\left(\lambda \right)$ in which $\lambda =N\mu_{0}^{2}/\sigma_{0}^{2}$. The cumulative distribution function is

(26) $$F_{Y_{k,m}}=1-Q_{\frac{1}{2}}\left(\sqrt{\lambda },\sqrt{x}\right)=1-\frac{1}{2}\left[erfc\left(\frac{\sqrt{x}+\sqrt{\lambda }}{\sqrt{2}}\right)+erfc\left(\frac{\sqrt{x}-\sqrt{\lambda }}{\sqrt{2}}\right)\right],$$

As a result, the expressions (8) and (9) for decoding common and private information for user *m* in the *k*-th user cluster can be re-expressed as

(27) $$\gamma_{k,m}^{c}=\frac{\tau_{k,m}a_{c}Y_{k,m}}{\tau_{k,m}\left(1-a_{c}\right)Y_{k,m}+{\left(d_{k,m}^{2}+L_{k}^{2}\right)}^{\alpha /2}},$$

(28) $$\gamma_{k,m}^{p}=\frac{\tau_{k,m}a_{k,m}^{p}Y_{k,m}}{\tau_{k,m}\left(1-a_{c}-a_{k,m}^{p}\right)Y_{k,m}+{\left(d_{k,m}^{2}+L_{k}^{2}\right)}^{\alpha /2}},$$

where $\tau_{k,m}=N\sigma_{0}^{2}\rho_{0}^{2}\beta_{k,m}p_{s}/\left(\sigma_{n}^{2}d_{AP,k}^{\alpha }\right)$. The ergodic rate for calculating the common and private information of user *m* in the *k*-th user cluster is given by

(29) $$\begin{array}{l} {\bar{R}}_{k,m}^{\chi }=E\left\{\log_{2}\left(1+\gamma_{k,m}^{\chi }\right)\right\}\\ =\frac{1}{\ln2}\int_{0}^{\infty }\frac{1-F_{k,m}^{\chi }\left(x\right)}{1+x}dx \end{array}.$$

Similar to the method of analyzing the ergodic rate of the system under discrete phase shifts, it can be further obtained that

(30) $${\bar{R}}_{k,m}^{\chi }=\frac{1}{\ln2}\int_{-\pi /2}^{\pi /2}\int_{\delta_{1}}^{\delta_{2}}\int_{0}^{\varpi_{k,m}^{\chi }}\frac{1-F_{Y_{k,m}}\left({Τ}_{k,m}^{\chi }\left(x\right)\Psi \left(y\right)\right)}{1+x}f_{d_{k,m}}\left(y\right)f_{\vartheta_{k,m}}\left(z\right)dxdydz,$$

where ${Τ}_{k,m}^{\chi }\left(x\right)=x/\left(v_{k,m}^{\chi }-u_{k,m}^{\chi }x\right)$ and $\Psi \left(y\right)={\left(y^{2}+L_{k}^{2}\right)}^{\alpha /2}$. For the reflecting user, $\delta_{1}=0$ and $\delta_{2}=R_{1}$. The expressions for the common information rate are $v_{k,r}^{c}=\tau_{k,r}a_{c}$, $u_{k,r}^{c}=\tau_{k,r}\left(1-a_{c}\right)$ and $\varpi_{k,r}^{c}=a_{c}/\left(1-a_{c}\right)$. The expressions for the private information rate are $v_{k,r}^{p}=\tau_{k,r}a_{k,r}^{p}$, $u_{k,r}^{p}=\tau_{k,r}\left(1-a_{c}-a_{k,r}^{p}\right)$ and $\varpi_{k,r}^{c}=a_{k,r}^{p}/\left(1-a_{c}-a_{k,r}^{p}\right)$. For the transmitting user, $\delta_{1}=R_{1}$ and $\delta_{2}=R_{2}$. The common information rate is calculated as $v_{k,t}^{c}=\tau_{k,t}a_{c}$, $u_{k,t}^{c}=\tau_{k,t}\left(1-a_{c}\right)$ and $\varpi_{k,t}^{c}=a_{c}/\left(1-a_{c}\right)$, and the private information rate is calculated as $v_{k,t}^{p}=\tau_{k,t}a_{k,t}^{p}$, $u_{k,t}^{p}=\tau_{k,t}\left(1-a_{c}-a_{k,t}^{p}\right)$, and $\varpi_{k,r}^{c}=a_{k,t}^{p}/\left(1-a_{c}-a_{k,t}^{p}\right)$. The expression of the ${\bar{R}}_{k,m}^{\chi }$ is

(31) $$\begin{array}{l} {\bar{R}}_{k,m}^{\chi }=\frac{1}{\ln2}\int_{\delta_{1}}^{\delta_{2}}\int_{0}^{\varpi_{k,m}^{\chi }}\frac{1-F_{Y_{k,m}}\left({Τ}_{k,m}^{\chi }\left(x\right)\Psi \left(y\right)\right)}{1+x}f_{d_{k,m}}\left(y\right)dxdy\\ =\frac{1}{\ln2}\int_{\delta_{1}}^{\delta_{2}}\int_{0}^{\varpi_{k,m}^{\chi }}\frac{1-F_{Y_{k,m}}\left({Τ}_{k,m}^{\chi }\left(x\right)\Psi \left(y\right)\right)}{1+x}f_{d_{k,m}}\left(y\right)dxdy \end{array}.$$

Substitute Equation (26) into (31) to obtain

(32) $${\bar{R}}_{k,m}^{\chi }=\frac{1}{\ln2\left(\delta_{2}^{2}-\delta_{1}^{2}\right)}\int_{\delta_{1}}^{\delta_{2}}\int_{0}^{\varpi_{k,m}^{\chi }}\frac{y\left[erfc\left(\frac{\sqrt{{Τ}_{k,m}^{\chi }\left(x\right)\Psi \left(y\right)}+\sqrt{\lambda }}{\sqrt{2}}\right)+erfc\left(\frac{\sqrt{{Τ}_{k,m}^{\chi }\left(x\right)\Psi \left(y\right)}-\sqrt{\lambda }}{\sqrt{2}}\right)\right]}{\left(1+x\right)}dxdy.$$

The inner integrals are permuted and processed using the Chebyshev–Gauss quadrature formula, which provides

(33) $$\begin{array}{l} \frac{\varpi_{k,m}^{\chi }}{2}\int_{-1}^{1}\frac{y\left[erfc\left(\frac{\sqrt{{Τ}_{k,m}^{\chi }\left(\varpi_{k,m}^{\chi }\left(x+1\right)/2\right)\Psi \left(y\right)}+\sqrt{\lambda }}{\sqrt{2}}\right)+erfc\left(\frac{\sqrt{{Τ}_{k,m}^{\chi }\left(\varpi_{k,m}^{\chi }\left(x+1\right)/2\right)\Psi \left(y\right)}-\sqrt{\lambda }}{\sqrt{2}}\right)\right]}{\left(1+\varpi_{k,m}^{\chi }\left(x+1\right)/2\right)}dxdy\\ =\frac{\varpi_{k,m}^{\chi }\pi }{2N_{p}}\sum_{i=1}^{N_{p}}\frac{y\sqrt{1-v_{i}^{2}}}{\left(1+\varpi_{k,m}^{\chi }\left(v_{i}+1\right)/2\right)}\left[erfc\left(\frac{\sqrt{{Τ}_{k,m}^{\chi }\left(\varpi_{k,m}^{\chi }\left(v_{i}+1\right)/2\right)\Psi \left(y\right)}+\sqrt{\lambda }}{\sqrt{2}}\right)\right.+\\ \left.erfc\left(\frac{\sqrt{{Τ}_{k,m}^{\chi }\left(\varpi_{k,m}^{\chi }\left(v_{i}+1\right)/2\right)\Psi \left(y\right)}-\sqrt{\lambda }}{\sqrt{2}}\right)\right] \end{array}.$$

Then, ${\bar{R}}_{k,m}^{\chi }$ can be re-expressed as

(34) $$\begin{array}{l} {\bar{R}}_{k,m}^{\chi }=\frac{\varpi_{k,m}^{\chi }\pi }{2\ln2\left(\delta_{2}^{2}-\delta_{1}^{2}\right)N_{p}}\times \\ \sum_{i=1}^{N_{p}}\underset{{Κ}_{1}}{\underset{︸}{\int_{\delta_{1}}^{\delta_{2}}\frac{y\sqrt{1-v_{i}^{2}}}{\left(1+\varpi_{k,m}^{\chi }\left(v_{i}+1\right)/2\right)}\left[erfc\left(\frac{\sqrt{{Τ}_{k,m}^{\chi }\left(\varpi_{k,m}^{\chi }\left(v_{i}+1\right)/2\right)\Psi \left(y\right)}+\sqrt{\lambda }}{\sqrt{2}}\right)+erfc\left(\frac{\sqrt{{Τ}_{k,m}^{\chi }\left(\varpi_{k,m}^{\chi }\left(v_{i}+1\right)/2\right)\Psi \left(y\right)}-\sqrt{\lambda }}{\sqrt{2}}\right)\right]dy}} \end{array}.$$

After integrating ${Κ}_{1}$ by permutation, the term ${Κ}_{1}$ in the above Equation (34) can be re-written as

(35) $$\begin{array}{l} {Κ}_{1}=\int_{\delta_{1}}^{\delta_{2}}\frac{y\sqrt{1-v_{i}^{2}}}{\left(1+\varpi_{k,m}^{\chi }\left(v_{i}+1\right)/2\right)}\left[erfc\left(\frac{\sqrt{{Τ}_{k,m}^{\chi }\left(\varpi_{k,m}^{\chi }\left(v_{i}+1\right)/2\right)\Psi \left(y\right)}+\sqrt{\lambda }}{\sqrt{2}}\right)\right.\\ \left.+erfc\left(\frac{\sqrt{{Τ}_{k,m}^{\chi }\left(\varpi_{k,m}^{\chi }\left(v_{i}+1\right)/2\right)\Psi \left(y\right)}-\sqrt{\lambda }}{\sqrt{2}}\right)\right]dy\\ =\frac{\delta_{2}-\delta_{1}}{2}\int_{-1}^{1}\left\{\frac{\left(\left(\delta_{2}-\delta_{1}\right)t/2+\left(\delta_{2}+\delta_{1}\right)/2\right)\sqrt{1-v_{i}^{2}}}{\left(1+\varpi_{k,m}^{\chi }\left(v_{i}+1\right)/2\right)}\times \right.\\ \left[erfc\left(\frac{\sqrt{{Τ}_{k,m}^{\chi }\left(\varpi_{k,m}^{\chi }\left(v_{i}+1\right)/2\right)\Psi \left(\left(\delta_{2}-\delta_{1}\right)t/2+\left(\delta_{2}+\delta_{1}\right)/2\right)}+\sqrt{\lambda }}{\sqrt{2}}\right)+\right.\\ \left.\left.erfc\left(\frac{\sqrt{{Τ}_{k,m}^{\chi }\left(\varpi_{k,m}^{\chi }\left(v_{i}+1\right)/2\right)\Psi \left(\left(\delta_{2}-\delta_{1}\right)t/2+\left(\delta_{2}+\delta_{1}\right)/2\right)}-\sqrt{\lambda }}{\sqrt{2}}\right)\right]\right\}dy \end{array}.$$

Next, the Chebyshev–Gauss quadrature formula can be used to obtain the approximate closed-form solution as

(36) $$\begin{array}{l} {Κ}_{1}=\frac{\left(\delta_{2}-\delta_{1}\right)\pi }{2N_{p}}\sum_{j=1}^{N_{p}}\left\{\frac{\left(\left(\delta_{2}-\delta_{1}\right)v_{j}/2+\left(\delta_{2}+\delta_{1}\right)/2\right)\sqrt{1-v_{i}^{2}}\sqrt{1-v_{j}^{2}}}{\left(1+\varpi_{k,m}^{\chi }\left(v_{i}+1\right)/2\right)}\times \right.\\ \left[erfc\left(\frac{\sqrt{{Τ}_{k,m}^{\chi }\left(\varpi_{k,m}^{\chi }\left(v_{i}+1\right)/2\right)\Psi \left(\left(\delta_{2}-\delta_{1}\right)v_{j}/2+\left(\delta_{2}+\delta_{1}\right)/2\right)}+\sqrt{\lambda }}{\sqrt{2}}\right)+\right.\\ \left.\left.erfc\left(\frac{\sqrt{{Τ}_{k,m}^{\chi }\left(\varpi_{k,m}^{\chi }\left(v_{i}+1\right)/2\right)\Psi \left(\left(\delta_{2}-\delta_{1}\right)v_{j}/2+\left(\delta_{2}+\delta_{1}\right)/2\right)}-\sqrt{\lambda }}{\sqrt{2}}\right)\right]\right\} \end{array},$$

where $v_{i}=\cos\left(\frac{2i-1}{2N_{p}}\pi \right)$ and $v_{j}=\cos\left(\frac{2j-1}{2N_{p}}\pi \right)$.

Substituting the expression for ${Κ}_{1}$ into Equation (34) leads to the approximate closed expression of ${\bar{R}}_{k,m}^{\chi }$ as

(37) $$\begin{array}{l} {\bar{R}}_{k,m}^{\chi }=\frac{\varpi_{k,m}^{\chi }\pi^{2}}{4\ln2\left(\delta_{2}+\delta_{1}\right)N_{p}^{2}}\sum_{i=1}^{N_{p}}\sum_{j=1}^{N_{p}}\left\{\frac{\left(\left(\delta_{2}-\delta_{1}\right)v_{j}/2+\left(\delta_{2}+\delta_{1}\right)/2\right)\sqrt{1-v_{i}^{2}}\sqrt{1-v_{j}^{2}}}{\left(1+\varpi_{k,m}^{\chi }\left(v_{i}+1\right)/2\right)}\times \right.\\ \left[erfc\left(\frac{\sqrt{{Τ}_{k,m}^{\chi }\left(\varpi_{k,m}^{\chi }\left(v_{i}+1\right)/2\right)\Psi \left(\left(\delta_{2}-\delta_{1}\right)v_{j}/2+\left(\delta_{2}+\delta_{1}\right)/2\right)}+\sqrt{\lambda }}{\sqrt{2}}\right)+\right.\\ \left.\left.erfc\left(\frac{\sqrt{{Τ}_{k,m}^{\chi }\left(\varpi_{k,m}^{\chi }\left(v_{i}+1\right)/2\right)\Psi \left(\left(\delta_{2}-\delta_{1}\right)v_{j}/2+\left(\delta_{2}+\delta_{1}\right)/2\right)}-\sqrt{\lambda }}{\sqrt{2}}\right)\right]\right\} \end{array}.$$

Thus, the sum ergodic rate under discrete and continuous phase shift systems is expressed as

(38) $${\bar{R}}_{tot}=\min\left\{{\bar{R}}_{k,m}^{c}\right\}+\sum_{k=1}^{K}\left({\bar{R}}_{k,t}^{p}+{\bar{R}}_{k,r}^{p}\right).$$

## 4. Simulation and Analysis

This paper analyzes the sum ergodic rate of an STAR-RIS-assisted RSMA downlink communication model under both discrete and continuous phase shift conditions. It also compares the impact of different factors on system performance and verifies the correctness of the ergodic rate derivation through Monte Carlo simulations. In this model, users are clustered at the transmitter, and their information is processed based on RSMA. Each user cluster is served by an STAR-RIS, and the mutual influence between different user clusters is not considered.

For simulation, six users and three user clusters are considered. It is assumed that the position of the AP with respect to each STAR-RIS is known and the distances between the AP and the STAR-RIS are $d_{1}=30$ m, $d_{2}=40$ m, and $d_{3}=50$ m. The same distribution of user positions is considered in each user cluster, which is $R_{1}=10$ m and $R_{2}=20$ m. The order of the Gaussian–Chebyshev polynomials $N_{p}$ is 50, and the number of Monte Carlo simulation times is set to $10^{6}$. The defaults for the rest of the simulation parameters are shown in Table 1.

**Figure 2:**
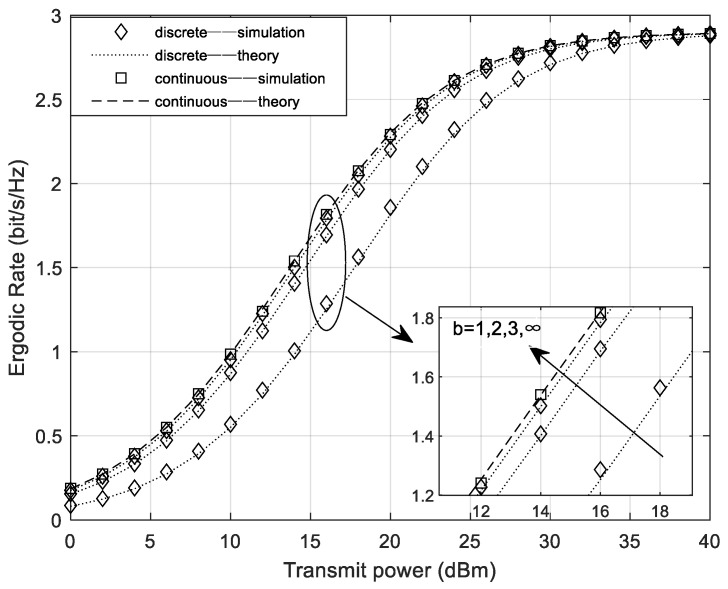
The system ergodic rate under different numbers of phase shift quantization bits.

Figure 2 shows the curves of system’s sum ergodic rate under different numbers of quantization bits and continuous phase shifts. In the simulation, the power ratio allocated to the common information part is set by default to $a_{c}=0.6$, and the remaining transmit power is equally divided among the private information parts of all users. From the figure, it can be observed that the system ergodic rate at b=1 is much lower than that at b=2 and b=3. Additionally, it can be seen that when b=3, the system ergodic rate is already very close to the ergodic rate under continuous phase shifts. Therefore, we can conclude that when designing quantized phase shifts, the number of quantization bits only needs to reach 3 to achieve a rate performance close to that of continuous phase shifts.

**Figure 3:**
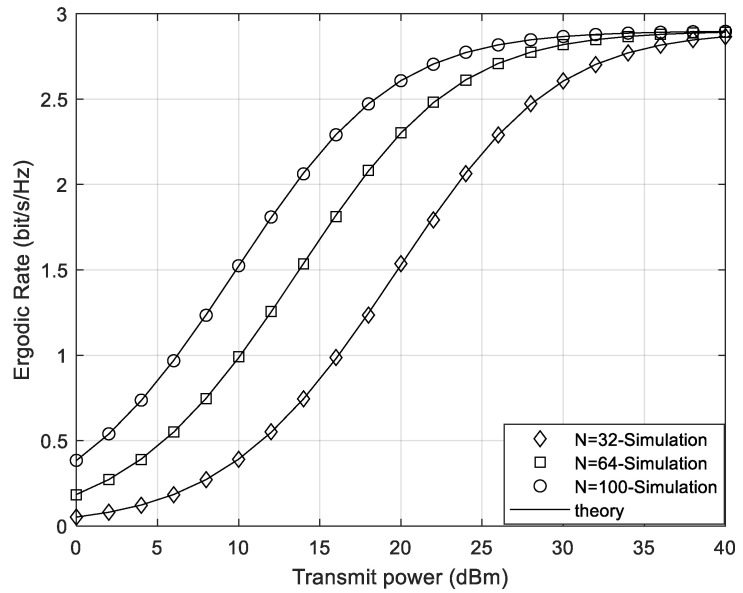
The system ergodic rate of different element numbers under continuous phase shifts.

**Figure 4:**
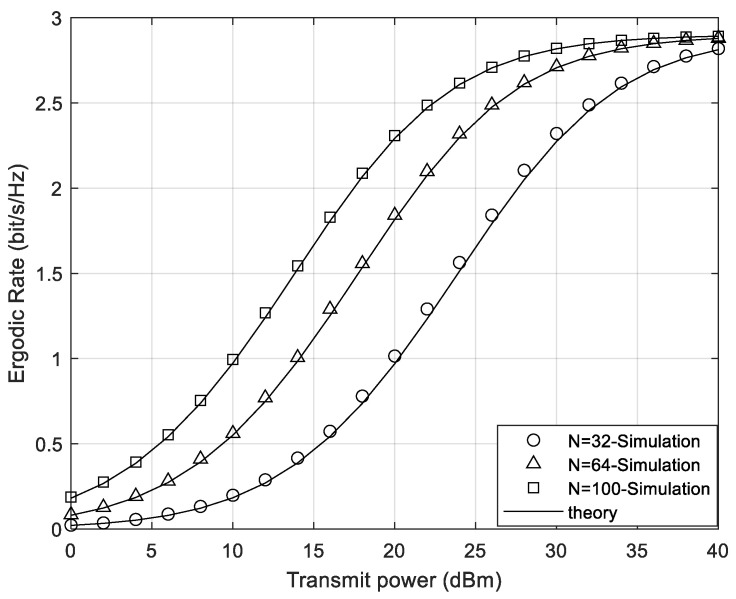
The system ergodic rate of different element numbers under discrete phase shifts.

Figure 3 and Figure 4 compare the ergodic rate under different numbers of STAR-RIS elements in the cases of continuous phase shifts and discrete phase shifts, respectively. As shown in Figure 3 and Figure 4, the theoretical curves of the system ergodic rate match well with the simulation curves. In both cases, as the number of elements increases, the total ergodic rate of the system increases accordingly. Thus, the more elements the STAR-RIS has, the greater the system gain it can bring. Additionally, since the impact of noise on the system can be ignored under high SNR, the system rate is a constant and is independent of the number of STAR-RIS elements under high SNR. Hence, the gain effect of STAR-RIS is more significant under the condition of low transmit power at the transmitter.

**Figure 5:**
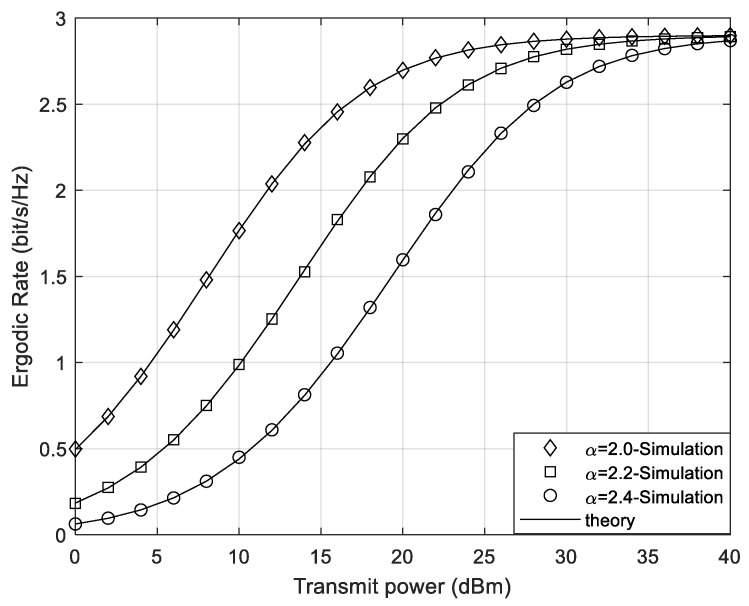
The system ergodic rate of different path loss exponents under continuous phase shifts.

**Figure 6:**
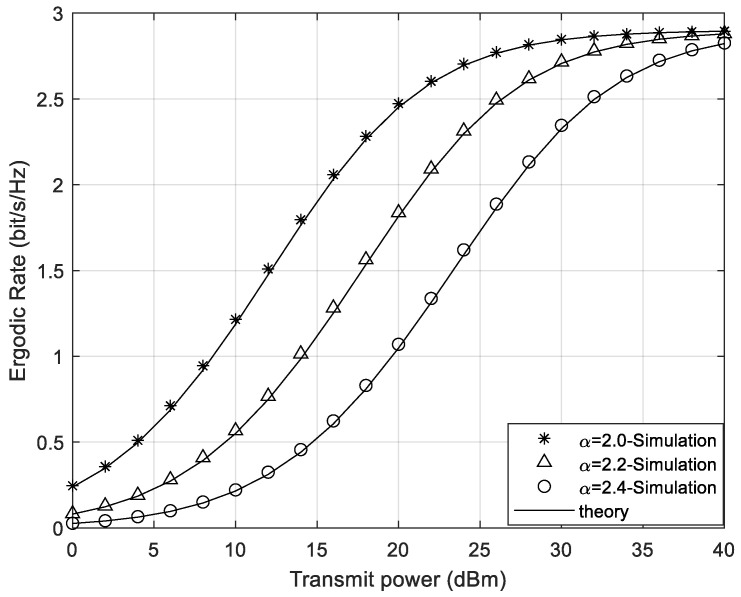
The system ergodic rate of different path loss exponents under discrete phase shifts.

In Figure 5 and Figure 6, under the conditions of continuous phase shifts and discrete phase shifts of STAR-RIS, the value of the path loss exponent α is changed, with α taking values of 2.0, 2.2, and 2.4, respectively. It can be seen that the ergodic rate for the system increases as the pass loss exponent decreases. This occurs because, with the communication distance and reference path loss remaining unchanged, a smaller α results in higher channel gain of the Rayleigh channel, which in turn leads to better channel quality. Thus, the user’s receiving end in this channel has a higher SINR. From both Figure 5 and Figure 6, it can be seen that regardless of whether the phase shifts are discrete or continuous, minimizing the path loss exponent as much as possible will bring better performance gains to the system. Additionally, under low transmit power conditions, reducing the path loss exponent results in a more significant increase in the ergodic rate of the STAR-RIS continuous phase shift system.

**Figure 7:**
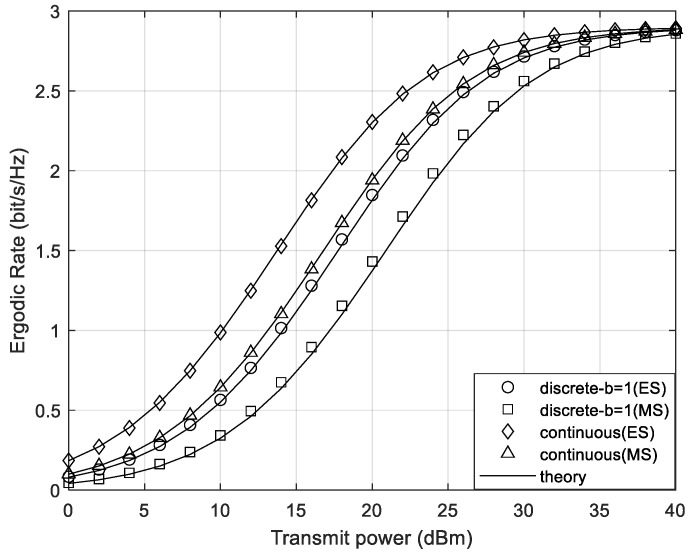
The system ergodic rate of different transmission modes.

Figure 7 compares the total system ergodic rate for both STAR-RIS ES and MS modes with continuous and discrete phase shifts. To ensure fairness, the MS transmission protocol has N/2 elements for transmission and reflection, respectively. From the figure, it can be seen that the total ergodic rate in the ES mode is higher than that in the MS mode for both discrete phase shift and continuous phase shift cases. This is due to the fact that only subsets of the elements are selected for transmission and reflection, respectively, in the MS mode, and the amplitude coefficients for transmission and reflection are limited to binary values. As a result, MS is usually unable to achieve the same full-dimensional transmission and reflection beamforming gains as ES. It is evident that the ergodic sum rate in the MS mode with quantization bit b=1 is lower than the ergodic sum rate with continuous phase shifts, which is consistent with the findings in Figure 2.

## 5. Conclusions

This paper mainly focuses on the analysis and comparison of the ergodic rate and performance of an STAR-RIS-assisted RSMA system under discrete and continuous phase shifts of STAR-RIS. Firstly, users are clustered at the transmitter, ensuring no interference between different user clusters. Additionally, it is considered that users are uniformly distributed within the range centered on the STAR-RIS. Secondly, the cumulative distribution function of the users’ SINR is derived based on the central limit theorem, and the approximate closed-form expression of the ergodic rate is obtained using Gaussian–Chebyshev integration. Monte Carlo simulations are conducted to show that the theoretical curves match the simulation curves, further validating the rationality and correctness of the theoretical derivation. During the simulations, by comparing the system performance under different phase shift quantization bits, it is concluded that to achieve performance close to the ideal continuous phase shift, the number of quantization bits for the phase shift should not be less than 3. Apart from this, it is found that increasing the number of elements and reducing the path loss exponent can improve the system performance to a certain extent.

## Figures and Tables

**Table 1:** Simulation parameters set based on discrete and continuous phase shifts of the STAR-RIS-assisted RSMA multi-user system.

Parameters	Default Value	Parameters	Default Value
number of STAR-RIS elements	N=64	noise power	$\sigma_{n}=-80$ dBm
path loss exponent	$\alpha_{0}=2.2$	path loss at a reference unit distance	$\rho_{0}=-30$ dB
number of quantization bits	b=1	amplitude coefficient of STAR-RIS	$\beta_{r}=\beta_{t}=0.5$

## Data Availability

The data presented in this study are available on request from the corresponding author.

## Abbreviations

Explanations of meaning of parameters used in the paper.

**Parameters**	**Explanations**
${\mathbb{C}}^{A\times B}$	complex matrices of dimension A×B
$CN\left(a,b\right)$	complex Gaussian distribution with mean a and variance b
$\left|\cdot \right|$	absolute value or modulus of a complex number or number of elements of a set
$diag\left\{x\right\}$	diagonal matrix with diagonal elements as vectors x
$E\left\{\cdot \right\}$	average value of a variable
$Sa\left(\cdot \right)$	sampling function
$\gamma \left(s,x\right)$	lower incomplete gamma function
$\Gamma \left(\cdot \right)$	gamma function
$Q_{M}\left(t,s\right)$	Markum Q Function
$erfc\left(\cdot \right)$	error complementarity function
